# Health economic studies of antimicrobial stewardship programmes: A scoping review

**DOI:** 10.3310/nihropenres.13726.1

**Published:** 2024-12-18

**Authors:** Pamela Nayyar, Celia Brown, Luiz Andrade, Richard Lilford

**Affiliations:** 1 University of Birmingham, Birmingham, England, B15 2TT, UK; 2 University of Warwick, Coventry, CV4 7AL, UK

**Keywords:** Antimicrobial stewardship programme, cost, cost effectiveness, cost utility, economic evaluation, antibiotic, scoping review, antimicrobial stewardship

## Abstract

**Aims:**

To conduct a scoping review of health economic evaluations of antimicrobial stewardship programmes (ASP). Our purpose was to summarise findings and to review different approaches taken.

**Methods:**

We reviewed economic evaluation studies retrieved from a number of sources, assessing the costs and effects of ASP. We described and synthesised data from studies published between 2002 and 2023 that included measures of both costs and effects/benefit of interventions.

**Results:**

Eight studies met the inclusion criteria. Six studies estimated cost-effectiveness, and two studies assessed cost utility. We found no cost-benefit studies. One of the studies was based on a randomised controlled trial. None of the studies took a broad perspective to include societal benefits that might arise from less resistant organisms on the environment contingent on reductions of prescriptions of broad-spectrum antibiotics.

**Conclusion:**

Limited evidence on the cost-effectiveness of Antimicrobial Stewardship Interventions studies suggests that the implementation of strategies to reduce antimicrobial resistance is worth the investment. However, producing a summary measure of ASP interventions is limited not just by the paucity of studies, but also heterogeneity of intervention types, variation in the implementation contexts and different methodological approaches.

## Plain Language Summary

This article is a scoping review of the health economic studies of antimicrobial stewardship programmes (ASP). Antimicrobial stewardship programmes aim to promote the careful use of antibiotics. This can be by reducing the amount of antibiotic prescribed; reducing the number of days the antibiotic is taken; or changing the antibiotic to a more suitable one. Another way is changing how the antibiotic is taken, from being injected into a vein to being taken as a pill. Antimicrobial stewardship interventions have the potential to reduce death and long-term illness by improving antibiotic selection; and to decrease the likelihood of organisms developing resistance to antibiotics. Ideally, health economic analysis should consider the effects of improved antibiotic stewardship on both the individual patient and the benefit to the wider population from less resistant bacteria in the environment. However, measuring the potential benefits of a reduction in resistant bacteria is difficult because any changes observed from a single ASP are small, relative to all the other factors at play. Our scoping review aimed to provide updated evidence for future antimicrobial stewardship programmes. We found six cost-effectiveness studies and two cost-utility studies. The studies use different methods for the economic analysis and collected data from different sources. The studies were also based around different clinical settings, had different types of intervention types and were undertaken in different types of institution in different countries. The vast differences in the studies included in the review suggest a strong argument for the standardisation of methods in terms of outcomes and cost measures.

## Introduction

Inappropriate use of antibiotics is highly undesirable. First, it leads to the avoidable emergence of resistant organisms that spread in the environment creating a major public health crisis. Second, it leads to sub-optimal clinical care since broad-spectrum antibiotics destroy normal commensal bacteria leading to an increase in super infections, of which *Clostridioides difficile* Infection (CDI) is the most important ^1, 2^. In addition, inappropriate prescribing may result in a mismatch between antibiotic and type of infection, thereby prolonging ill-health or even leading to death.

To control this problem, numerous antimicrobial stewardship programmes (ASP) have been developed. We conceptualize ASPs as interventions to make use of existing knowledge to cause hospital staff to ’de-escalate’ antibiotics by: stopping antibiotics or reducing their dosage/duration; changing from intravenous to oral administration; or changing to a more suitable antibiotic (including from a broad to a narrow spectrum antibiotic).

ASP interventions have two distinct types of potential benefit. First, they benefit the individual patient, by reducing opportunistic infections (especially CDI) and by improving antibiotic selection, thereby decreasing mortality and morbidity. Second, the prevalence of resistant organisms in the broader environment should gradually decline, protecting the population as a whole. Ideally, health economic analysis should consider the effects of improved antibiotic stewardship on both the individual patient and the societal benefit from less resistant bacteria in the environment. However, measuring the potential benefits of reducing the environmental burden of resistant bacteria is difficult because the plausible proportionate change in outcomes from a single ASP are small, relative to all the other factors at play.

An immediate target of ASPs is to change (often reduce) the amount of antibiotic prescribed. However, there are many different types of antibiotics, dosages, and routes of administration. For this reason, a summary metric has been derived: the defined daily dose (DDD). This is defined as the average dose per day for a specific drug used for its main indication in adults ^3^.

The literature on the evaluation of ASP is relatively abundant. In studies conducted in the last 20 years, Nathwani *et al.* ^4^ conducted a systematic review of antibiotic stewardship programs and reported decreases in antibiotic usage (measured in DDD) ranging from -6% ^5^ to – 55% ^6^. Reductions in antimicrobial costs from -12% ^7^ to -69% ^8^ were also reported in the review. Trotter *et al.*’s systematic review found that ASPs led to decreases in antimicrobial consumption and cost, with a reduction in DDD in studies utilising and audit and feedback approach ^9^. With regards to CDI, Baur *et al.* ^10^ conducted a systematic review and meta-analysis of studies investigating the impact of ASP on CDI and found an overall reduction of 32% in infections associated with *Clostridioides difficile,* although, the studies included in this review were conducted when baseline rates of CDI (and therefore the headroom for improvement) were considerably higher than they are now. In the systematic review of ASPs for surgical site infections by Martinez-Sobalvarro *et al.* ^11^ some of the studies that did not monetise patient benefit were nevertheless called cost-benefit. For example, Zhou *et al.* ^12^ related cost savings from changes in antibiotic use to the cost of the intervention in terms of pharmacist time but did not value the health benefits of the intervention. There are many other studies, such as Wang *et al.* ^13^, that looked only at costs of antibiotics with estimates of savings, without detailing intervention costs.

Here we report on a scoping review of the literature of health economic studies of antibiotic stewardship programmes with the following objectives:

- Determine the type of data that have been collected.
- Determine the types of economic model used.
- Examine the perspectives taken.
- Better understand methodological issues presented in this field.
- Assemble information on the estimates of cost-effectiveness.
- Provide guidance to future projects aiming to estimate the value for money of digital ASP.

We planned to include:

1. Simple measures of cost per unit of clinical effectiveness
2. Cost-utility
3. Cost-benefit, where monetary values are ascribed to improved clinical outcomes

We did not include studies based on therapeutic drug monitoring or complex drug monitoring regimes designed to avoid toxicity while maintaining effective antibiotic concentrations ^14^.

## Methods

### Patient and Public Involvement

Patient partners were not involved in the design of this scoping review.

### Search strategy

We searched for health economic studies of antimicrobial stewardship programmes using the databases PubMed, EconLit, Business Source Alumni Edition, Business Source Premier and CINAHL Pluss. Our search strings included the following terms: ((cost-effectiveness) OR (cost-benefit) OR (cost-utility)) AND ((antimicrobial stewardship) OR (antibiotic stewardship)) AND ((hospital) OR (secondary care)). Search and selection of studies was carried out between April and May 2022 and October 2023 (in PubMed). We also conducted a hand search of studies by screening the references of studies retrieved through the above research strings.

### Eligibility criteria

We limited our review to studies conducted within a hospital setting and published in English between January 2002 and October 2023. We selected studies measuring *both* costs and clinical effectiveness of the intervention. Cost measures had to include the intervention cost and could include in addition, antibiotic costs or hospital costs. We selected only studies that included a valued (i.e. clinical) outcome (death, length of stay, *Clostridioides difficile* infection), excluding studies that only mention process (mediating) outcomes such as DDD and total antibiotic use. We excluded antifungal stewardship programmes and studies using diagnostic test innovations as a mechanism to improve antibiotic utilisation (e.g., procalcitonin tests and rapid diagnosis tests).

### Study selection

The titles and abstracts of studies returned by our database were searched to select potentially eligible studies. We retrieved and scrutinised those studies to make our final selection. Full texts were scanned by LA and PN.

### Data extraction

Papers would be categorised into three groups based on the method used: cost-effectiveness or cost benefit. To categorise papers based on these three different methods, we used the following definitions of economic evaluations, according to the definitions used by Shiell *et al.* ^15^ as follows:

1. Cost-effectiveness analysis: this method is used when the intervention is evaluated by measuring the effects in physical units of one health outcome (mortality, healthcare infections, adverse event rates). The cost of the intervention can then be used to calculate the incremental cost effectiveness ratio (ICER), such as the decrease in cost per death averted.
2. Cost-utility analysis: intervention costs are compared to health outcomes expressed in quality adjusted life years (QALYs), disability adjusted life years (DALYs) or healthy years equivalents (HYEs) to enable comparisons between treatments for different diseases.
3. Cost-benefit analysis: all costs and benefits of the intervention are defined and quantified in monetary values. The incremental benefit-cost measure is expressed in gain/return in monetary units per unit of cost (e.g., per GBP or USD).

No costs were reported in UK pounds sterling. To enable consistency and fair comparisons between studies, we firstly converted costs to pounds sterling using the exchange rate at 30 June in the year of costing ( https://www.xe.com/currencytables/). Secondly, costs in GBP were inflated to 2021/22 values using the NHSCII pay & prices index; the 2022/23 index was not available at the time of writing, so the 2021/22 indices were re-used to deflate costs obtained at 2022/23 prices ( https://www.pssru.ac.uk/unitcostsreport/) ^16^. For costs in 2018, we started at the 2018/19 NHSCII value, for example. We report base-case results as provided by the authors.

## Results

### Articles reviewed

**Figure 1.**
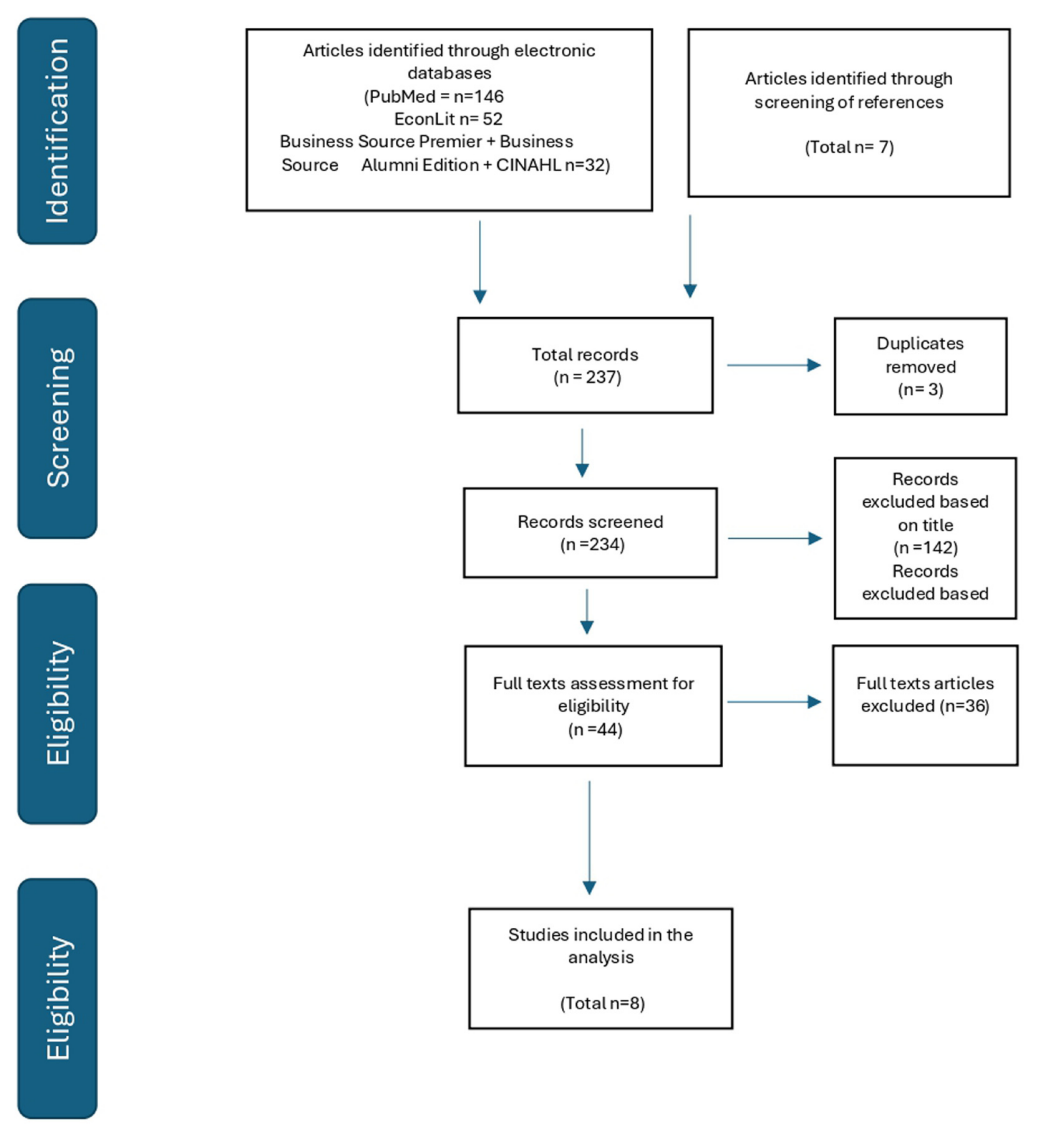
PRISMA diagram of the selection of economic evaluation studies of ASP.

Two hundred and twenty-eight articles were returned using the search strings described above and the further search for eligible studies identified amongst the references returned seven articles to give a total of 237 studies ( Figure 1).

One hundred and ninety of the two hundred and thirty-seven studies were rejected after examination of title and abstract, leaving forty-four studies for full text assessment. Note, that in line with our stated aims, we only included studies that provided intervention costs *and* a summary measure of effectiveness or utility/benefit. This means that we did not include studies such as Mahmoudi *et al.* ^17^ and Alawi *et al.* ^18^ that did not specify the costs of the intervention. Eight studies ( Table 1 and Table 2) were then selected after reading the full articles ^19– 26^. Outcomes included CDI, length of stay (LOS) and mortality. Only one study was based on a Randomized Control Trial (RCT) ^21^. The other studies were observational studies – one of these based on secondary data - and all took a health service perspective rather than a broader societal perspective. Thus, none of the studies attempted to capture the effects of payback that might occur to society more broadly as a result of lower level of resistant organisms in the environment. We return to the issue of potential societal types of paybacks in the discussion.

**Table 1.** Summary of costs and effectiveness parameters from the cost-effectiveness studies of antimicrobial stewardship programmes.

Study	Study Design (Country)	Data source	Intervention	Main Outcomes Cost-effectiveness ratios in 2021/22 GBP
Okumura *et al.* (2016) *Costs: 2013 USD*	Retrospective study (Brazil)	Primary research	Comparison between two ASP types: guidelines with pharmacist versus a bundled strategy of audit feedback and education	Cost per 30-day death averted (using Markov modelling results): Conventional ASP: 21,856 Bundled ASP: 20,731 ICER for bundled cf. conventional: 14,530
Ruiz-Ramos *et al.* (2017) *Costs: 2015 Euro*	Literature review and decision tree model (Spain)	Secondary research (data from published literature)	Antimicrobial restrictions + guidelines + formal re- assessment	Cost per avoided resistance: 5,881 Cost per life year gained: 7,840
van Daalen *et al.* (2017) *Costs: 2015 Euro*	Step-wedged cluster randomised trial (Netherlands)	Primary research data from nine hospitals	Antibiotic checklist	Cost per additional patient with appropriate treatment: 43
Slayton *et al.* (2015) *Costs: 2011 USD*	Literature review and database observational study and Markov model with a 5-year time horizon (USA)	CDC surveillance systems, the AHRQ Healthcare Cost and Utilization Project, and literature reviews	Guidelines and ASP personnel	Based on 50% effectiveness of the intervention: Cost saving per CDI case avoided: 3,700 Cost saving per CDI death averted: 23,000 *The intervention * *dominates*
Lin *et al.* (2013) *Costs: 2009 USD*	Before-after study (Taiwan)	Primary research data (retrospective chart review and sample survey)	ASP comprised systematic education on antimicrobial stewardship; pharmacists evaluating antimicrobial use; and the regular reporting of outcomes to all staff.	Cost saving per HAI avoided (estimated from data provided): 57,290 *The intervention * *dominates*
Zhou *et al.* (2023) *Costs: 2019 USD*	Matched case-control study (China)	Electronic hospital records	Pharmacist-led intervention	Cost saving per infection avoided (estimated from data provided): 980 Cost saving per *extra* readmission: 6,780

**Table 2.** Summary of costs and effectiveness parameters used in the cost-utility studies of antimicrobial stewardship programmes.

Study	Type of CEA	Data source	Type of ASP	Cost measures	Effectiveness parameters	Method	Results Cost-utility ratios in 2021/22 GBP
Scheetz *et al.* 2009 *Costs: 2008 USD*	Cost utility	Literature and expert opinion	AS team + Infectious disease (ID) specialist to better prescribe antibiotics (Structural)	Hospital costs Blood culture costs Costs of implementing a computerized clinical decision support (CDSS) tool	Quality-adjusted life years (QALYs)	Decision tree modelling (and probabilistic sensitivity analysis)	Cost per QALY gained: 1,496
Gebretekle *et al.* 2021 *Costs: 2018 USD*	Cost utility (lifetime time horizon)	Primary and published literature	Laboratory- supported and pharmacist- led AMS programme (Persuasive and structural)	All direct medical costs: medication cost, investigation/procedural cost, microbiology/culture and sensitivity test cost, staff time cost, admission and other hospitalisation costs.	Quality-adjusted life-years (QALYs)	Markov model	Saving per QALY gained: 1,729 *The * *intervention * *dominates*

### Types of study

We found six studies that fulfilled our criteria in the cost-effectiveness category and two in the cost-utility category. We found no cost-benefit studies as defined above (although we did find a cost-effectiveness study (Slayton *et al.* ^22^) that was described as a cost-benefit study by the authors).

### Cost-effectiveness studies

The six studies that included the intervention cost and at least one clinical effect are described in Table 1. The table includes the single randomised trial recorded in our review ^21^. This was a step-wedged cluster trial conducted in nine hospitals. There were four observational studies and one purely literature-based study ^20^. The intervention dominated conventional care in two of the six studies ^22, 23^ i.e., it reduced overall costs net of the intervention cost *and* improved health outcomes. Four studies used number of infections as an outcome measure, but not consistently: for example, Slayton *et al.* ^22^ only considered CDI while Lin *et al.* ^23^ used all healthcare-associated infections (HAIs).

### Cost utility studies

The two cost-utility studies reported here are both associated with the use of ASP for treatment of bloodstream infections ( Table 2). Types of costs included in the analyses were hospital costs, blood culture costs, costs of implementing the computerized clinical decision system (CCDS) and staff time costs. The effectiveness parameter was measured as quality-adjusted life years (QALYs) in both studies. The intervention dominates the control in Gebretekle *et al.* ^25^, with a saving of £1,700 per QALY gained. The intervention reported by Scheetz *et al.* ^26^ was highly likely to be cost-effective, with a cost per QALY gained of £1,500.

## Discussion

### Literature

Our scoping review found six studies on the cost-effectiveness of ASPs and two cost-utility analyses. Many other papers included details of medication cost savings resulting from ASP implementation but did not measure clinical outcomes and/or did not provide details of intervention costs.

### Cost estimations

Hospitalisation costs in the form of bed days have a large impact on total costs and were used as an/the outcome in four of the studies ^19, 22, 25, 26^. Overall, the studies provided mixed results on the effect of ASPs on length of stay/readmission. This is mirrored by the broader literature. For example, while Hagert *et al.* ^27^ did not find any difference in length of stay for patients in the ASP compared to the control group, Niwa *et al.* ^7^ reported a reduction of one day in the average length of hospital stay following implementation of an ASP. A reduction in *Clostridioides difficile* infection rates, as a result of effective ASPs, will reduce hospitalisation costs and is likely to have major impact on the cost-effectiveness analysis. Many clinical studies have investigated the effects of CDI on hospital stay and results show that CDI is associated with longer length of stay and consequently is an important driver for hospital costs ^28^. Previous analysis of the effects of CDI on healthcare costs have shown that direct healthcare expenditures and opportunity costs associated with additional LOS were the main CDI cost drivers ^29^. One study included in this review specifically estimated the cost savings associated with the reduction of CDI rates ^22^.

Only one study captured the effects of early switch from intravenous to oral administration ^21^. It is worth noting that many authors have investigated the effects of early switching antibiotics from IV to parental administration, showing either positive outcomes for patients or decreasing healthcare costs ^30– 32^.

Implementation of ASP had cost implications for those in the workforce who are involved at the development, implementation, and operational levels. Two studies provided detailed information about the different types of health staff associated with the ASP intervention ^21, 22^. Van Daalen *et al.* ^21^, for instance, reported staff costs in detail, including activities of coordinator and local supervisor in planning and sharing materials with the team, giving briefings and travelling, along with physicians’ workloads (such as time specialists and residents spend attending briefings or performing e-learning). Workload of medical doctors in charge of implementing ASP is an important issue since increasing their responsibilities associated with the new intervention may affect the capacity of physicians to better prescribe, as showed in qualitative study conducted in India by Baubie *et al.* ^33^ Other studies provide scant detail on costs (e.g. Lin *et al.* ^23^), such that the costs provided are likely to be an underestimate of total costs, meaning that cost-effectiveness will be overestimated.

### Effectiveness measures

Several effectiveness outcomes were used in the economic evaluation studies retrieved for this scoping review. Two studies used mortality rates as an effectiveness measure ^19, 20^. One cost-effectiveness study used 30-day mortality rate as the main effectiveness outcome and DDD and resistant infection rates as secondary outcomes ^19^. Another study used CDI rates and potential additional deaths associated with CDI to estimate the cost per avoided resistance and cost-per-life-year gained (LYG) ^20^. The use of mortality rate to assess the quality of hospital care has been discussed in more detail elsewhere by Lilford and Pronovost ^34^ indicating that standardised mortality rates may not be reliable to judge the performance of healthcare services.

One study reported a reduction in HAI rates but an increase in readmission rates (although neither effect was statistically significant), alongside an overall cost saving, driven by a reduction in drug and antibiotic costs ^24^. Without converting the clinical effects to QALYs in a cost-utility analysis, it is not possible to determine whether the intervention has an overall positive or negative impact on health, or whether increasing readmissions is justified for achieving cost-savings.

The studies included here report on antimicrobial consumption and the clinical benefits that may flow from reduced iatrogenic infections, especially *Clostridioides difficile*. However, none of the interventions specifically considered the potential effect of ASPs in better targeting therapies as a result of Bug-Antibiotic Mismatch (BAM). ASPs have the potential to reduce mismatch by prompting clinicians to change the antibiotic prescription in response to results from the microbiological laboratory recorded in the electronic record.

Lastly, the conclusions of Slayton *et al.* ^22^ are likely to be optimistic when applied to an ASP alone since it was a multi-component intervention not limited to ASP.

### Societal perspective

As stated in the Introduction, society as a whole benefits from improved antibiotics prescribing. However, none of the studies captured the potential benefits and cost (savings) of reduced drug-resistant infections resulting from a reduced micro-biological resistance in the environment. We speculate that this is not because scholars have not considered this issue, but because of methodological difficulties. One barrier to such studies is the difficulty of estimating the contribution of improved prescribing in any domain (community or hospitals) to the overall burden of resistant organisms in the environment. It is also difficult to capture the burden of antibiotic prescribing on other hospitals, other health care settings and in veterinary and agricultural practice. We have identified a research group that is tackling this challenging problem ^35^.

### Economic models

The included studies use different methods for the economic analysis and collect data from distinct sources. Furthermore, they cover different clinical scenarios, very different intervention (ASP) types and different types of institution in different places. Faced with such massive heterogeneity of method, topic and context, and a limited number of studies, it would be inappropriate to draw general conclusions. Rather the papers are instructive of the many methodological challenges such as the choice of effectiveness parameters in analysing a service delivery intervention such as ASPs ^36^.

### Implications

The extreme heterogeneity of studies included in this review has a number of implications for future research. First, there is a strong argument for standardisation of outcomes (costs and effects) across methods in terms of costs and other outcomes included. We recommend that an international meeting be convened to see if such agreement can be forged. Second, it is unlikely to be sensible to combine effectiveness parameters for ASP interventions in a quantitative meta-analysis. It is difficult to see how one summary measure can inform a future decision notwithstanding random effects methods. If there were very large numbers of individual studies then it might be possible to subgroup by, for example, intervention type or to construct more complex multi-level meta-regression ^37^. Absent from such a large ensemble of studies, we do not think summary measures of effectiveness across studies are useful. Third, pending the evolution of a very large number of individual studies, we advocate the development of an agreed model relating intervention to outcomes. The individual decision makers could then populate such models with parameters estimates informed by literature but adapted to local circumstances. Such a model would also inform the mediating (process) and outcome variables that should be collated routinely.

### Limitations

A limitation of this scoping review is that only eight studies were identified for inclusion. Hence, any attempt to produce a summary measure of ASP interventions is limited by the paucity of studies, and heterogeneity of intervention types, variation in the implementation contexts and the different methodological approaches.

## Conclusion

Several challenges still persist in the implementation of economic evaluation of ASP and this study aimed to provide updated evidence and guidance for future antimicrobial stewardship programmes, especially those intending to implement software to support the intervention.

## Data Availability

No data are associated with this article. **Figshare:** PRISMA Checklist for “Health economic studies of antimicrobial stewardship programmes: A scoping review”, ***DOI. https://doi.org/10.6084/m9.figshare.27727389.v1***

## References

[1] Goossens H : Antibiotic consumption and link to resistance. *Clin Microbiol Infect.* 2009;15 Suppl 3(Suppl 3):12–5. 10.1111/j.1469-0691.2009.02725.x 19366364

[2] Coulter S Merollini K Roberts JA : The need for cost-effectiveness analyses of Antimicrobial Stewardship Programmes: a structured review. *Int J Antimicrob Agents.* 2015;46(2):140–9. 10.1016/j.ijantimicag.2015.04.007 26058776

[3] World Health Organization, Collaborating Centre for Drug Statistics Methodology: ATC Index with DDDs.Oslo, Norway WHO,2004; Accessed 30 November 2021. Reference Source

[4] Nathwani D Varghese D Stephens J : Value of hospital Antimicrobial Stewardship Programs [ASPs]: a systematic review. *Antimicrob Resist Infect Control.* 2019;8: 35. 10.1186/s13756-019-0471-0 30805182 PMC6373132

[5] Ng CK Wu TC Chan WM : Clinical and economic impact of an antibiotics stewardship programme in a regional hospital in Hong Kong. *Qual Saf Health Care.* 2008;17(5):387–92. 10.1136/qshc.2007.023267 18842981

[6] Leung V Gill S Sauve J : Growing a “positive culture” of antimicrobial stewardship in a community hospital. *Can J Hosp Pharm.* 2011;64(5):314–20. 10.4212/cjhp.v64i5.1065 22479082 PMC3203822

[7] Niwa T Shinoda Y Suzuki A : Outcome measurement of extensive implementation of antimicrobial stewardship in patients receiving intravenous antibiotics in a Japanese university hospital. *Int J Clin Pract.* 2012;66(10):999–1008. 10.1111/j.1742-1241.2012.02999.x 22846073 PMC3469737

[8] Magedanz L Silliprandi EM dos Santos RP : Impact of the pharmacist on a multidisciplinary team in an Antimicrobial Stewardship Program: a quasi-experimental study. *Int J Clin Pharm.* 2012;34(2):290–4. 10.1007/s11096-012-9621-7 22382886

[9] Trotter NE Slight SP Karimi R : The effect of digital Antimicrobial Stewardship Programmes on antimicrobial usage, length of stay, mortality and cost. *Inform Med Unlocked.* 2023;37: 101183. 10.1016/j.imu.2023.101183

[10] Baur D Gladstone BP Burkert F : Effect of antibiotic stewardship on the incidence of infection and colonisation with antibiotic-resistant bacteria and *Clostridium difficile* infection: a systematic review and meta-analysis. *Lancet Infect Dis.* 2017;17(9):990–1001. 10.1016/S1473-3099(17)30325-0 28629876

[11] Martinez-Sobalvarro JV Alves Pereira A Birges Pereira L : Antimicrobial stewardship for surgical antibiotic prophylaxis and surgical site infections: a systematic review. *Int J Clin Pharm.* 2022;44(2):301–319. 10.1007/s11096-021-01358-4 34843035

[12] Zhou L Ma J Gao J : Optimizing prophylactic antibiotic practice for cardiothoracic surgery by pharmacists’ effects. *Medicine (Baltimore).* 2016;95(9): e2753. 10.1097/MD.0000000000002753 26945362 PMC4782846

[13] Wang J Dong M Lu Y : Impact of pharmacist interventions on rational prophylactic antibiotic use and cost saving in elective cesarean section. *Int J Clin Pharmacol Ther.* 2015;53(8):605–15. 10.5414/CP202334 26104036

[14] Telles JP Morales R Jr Yamada CH : Optimization of antimicrobial stewardship programs using therapeutic drug monitoring and pharmacokinetics-pharmacodynamics protocols: a cost-benefit review. *Ther Drug Monit.* 2023;45(2):200–208. 10.1097/FTD.0000000000001067 36622029

[15] Shiell A Donaldson C Mitton C : Health economic evaluation. *J Epidemiol Community Health.* 2002;56(2):85–8. 10.1136/jech.56.2.85 11812804 PMC1732075

[16] Jones KC Weatherly H Birch S : Unit costs of health and social care 2022 manual.Technical report, Personal Social Services Research Unit (University of Kent) & Centre for Health Economics (University of York), Kent, UK,2023. 10.22024/UniKent/01.02.100519

[17] Mahmoudi L Sepasian A Firouzabadi D : The impact of an Antibiotic Stewardship Program on the consumption of specific Antimicrobials and their cost burden: a hospital-wide intervention. *Risk Manag Healthc Policy.* 2020;13:1701–1709. 10.2147/RMHP.S265407 33061704 PMC7520156

[18] Alawi MM Tashkandi WA Basheikh MA : Effectiveness of Antimicrobial Stewardship Program in Long-Term Care: a five-year prospective single-center study. *Interdiscip Perspect Infect Dis.* 2022;2022: 8140429. 10.1155/2022/8140429 35464254 PMC9019452

[19] Okumura LM Riveros BS Gomes-da-Silva MM : A cost-effectiveness analysis of two different Antimicrobial Stewardship Programs. *Braz J Infect Dis.* 2016;20(3):255–61. 10.1016/j.bjid.2016.02.005 27094234 PMC9425487

[20] Ruiz-Ramos J Frasquet J Romá E : Cost-effectiveness analysis of implementing an Antimicrobial Stewardship Program in critical care units. *J Med Econ.* 2017;20(6):652–659. 10.1080/13696998.2017.1311903 28345481

[21] van Daalen FV Opmeer BC Prins JM : The economic evaluation of an antibiotic checklist as Antimicrobial Stewardship Intervention. *J Antimicrob Chemother.* 2017;72(11):3213–3221. 10.1093/jac/dkx259 28981722

[22] Slayton RB Scott RD Baggs J : The cost-benefit of federal investment in preventing *Clostridium difficile* infections through the use of a multifaceted infection control and Antimicrobial Stewardship Program. *Infect Control Hosp Epidemiol.* 2015;36(6):681–7. 10.1017/ice.2015.43 25783204 PMC6550306

[23] Lin YS Lin IF Yen YF : Impact of an Antimicrobial Stewardship Program with multidisciplinary cooperation in a community public teaching hospital in Taiwan. *Am J Infect Control.* 2013;41(11):1069–1072. 10.1016/j.ajic.2013.04.004 23870295

[24] Zhou X Gong J Su D : Effect of pharmacist intervention on antibiotic prophylaxis in orthopedic internal fixation: a retrospective study. *Res Soc Adm Pharm.* 2023;19(2):301–307. 10.1016/j.sapharm.2022.10.002 36266174

[25] Gebretekle GB Mariam DH Mac S : Cost-utility analysis of Antimicrobial Stewardship Programme at a tertiary teaching hospital in Ethiopia. *BMJ Open.* 2021;11(12): e047515. 10.1136/bmjopen-2020-047515 34921071 PMC8685939

[26] Scheetz MH Bolon MK Postelnick M : Cost-effectiveness analysis of an Antimicrobial Stewardship Team on bloodstream infections: a probabilistic analysis. *J Antimicrob Chemother.* 2009;63(4):816–25. 10.1093/jac/dkp004 19202150

[27] Hagert BL Williams C Wieser CM : Implementation and outcome assessment of an inpatient Antimicrobial Stewardship Program. *Hosp Pharm.* 2012;47(12):939–45. 10.1310/hpj4712-939

[28] Brain DC Barnett AG Yakob L : Reducing length of stay to improve *Clostridium difficile*-related health outcomes. *Infect Dis Health.* 2018;23(2):87–92. 10.1016/j.idh.2018.01.001 38715308

[29] Ghantoji SS Sail K Lairson DR : Economic healthcare costs of *Clostridium difficile* infection: a systematic review. *J Hosp Infect.* 2010;74(4):309–18. 10.1016/j.jhin.2009.10.016 20153547

[30] Boclé H Lavigne JP Cellier N : Effectiveness of early switching from intravenous to oral antibiotic therapy in *Staphylococcus aureus* prosthetic bone and joint or orthopedic metalware-associated infections. *BMC Musculoskelet Disord.* 2021;22(1): 315. 10.1186/s12891-021-04191-y 33784991 PMC8008605

[31] Mertz D Koller M Haller P : Outcomes of early switching from intravenous to oral antibiotics on medical wards. *J Antimicrob Chemother.* 2009;64(1):188–99. 10.1093/jac/dkp131 19401304 PMC2692500

[32] Mouwen AMA Dijkstra JA Jong E : Early switching of antibiotic therapy from Intravenous to oral using a combination of education, pocket-sized cards and switch advice: a practical intervention resulting in reduced length of hospital stay. *Int J Antimicrob Agents.* 2020;55(1): 105769. 10.1016/j.ijantimicag.2019.07.020 31362046

[33] Baubie K Shaughnessy C Kostiuk L : Evaluating Antibiotic Stewardship in a tertiary care hospital in Kerala, India: a qualitative interview study. *BMJ Open.* 2019;9(5): e026193. 10.1136/bmjopen-2018-026193 31092653 PMC6530383

[34] Lilford R Pronovost P : Using hospital mortality rates to judge hospital performance: a bad idea that just won't go away. *BMJ.* 2010;340: c2016. 10.1136/bmj.c2016 20406861

[35] Jit M Ng DHL Luangasanatip N : Quantifying the economic cost of Antibiotic Resistance and the impact of related interventions: rapid methodological review, conceptual framework and recommendations for future studies. *BMC Med.* 2020;18(1): 38. 10.1186/s12916-020-1507-2 32138748 PMC7059710

[36] Sutton M Garfield-Birkbeck S Martin G : Economic analysis of service and delivery interventions in health care. *Health Soc Care Deliv Res.* Southampton, UK: NIHR Journals Library;2018;6(5). 10.3310/hsdr06050 29481019

[37] Gelman A Hill J : Data Analysis Using Regression and Multilevel/Hierarchical Models.Cambridge, UK: Cambridge University Press;2006. 10.1017/CBO9780511790942

