# Supplementary material for: Health economic studies of antimicrobial stewardship programmes: A scoping review
Source: NIHR Open Res. 2024 Dec 18;4:78. [Version 1] doi: 10.3310/nihropenres.13726.1 (PMC12933046; doi:10.3310/nihropenres.13726.1)
Supplement: Peer review history

# Reviewer report: Reviewer response for version 1

**DOI:** 10.3310/nihropenres.14905.r39465

**Published:** 2026-03-02

**Authors:** Jayashree Purkayastha

**Affiliations:** 1 Manipal Academy of Higher Education, Bengaluru, Karnataka, India

Summary

 This article is a scoping review of the health economic studies of antimicrobial stewardship programmes. Search was based on ASP/hospital based/secondary care. Only those studies were included which provided intervention costs and measure of effectiveness or utility/benefit.

 Major points: The 8 studies included ranged from LMIC to developed countries which were different in their economic status and per capita income and methods of intervention were different however authors projected the main outcomes, the cost effectiveness and cost utility of the studies which is the  main strength of the Review.

 Minor points: The authors provided study design, country and interventions in the 6 cost effectiveness studies which is a positive point of the review. However in the 2 cost utility studies cost measures and methods were described but country of the study was not mentioned. Authors can mention the country also.

 These are the strength of the study.

 Limitations:  It was not mentioned whether the 8 studies included were adult oriented or Pediatrics. Why only secondary care was included and not tertiary care needs to be explained. Quality of the 8 studies were not analysed by CHEERS criteria.

 Implications for future research: In  this para authors have suggested that their studies were extreme heterogenous hence standardization of outcomes(cost and effects) should be done in future. However I would suggest that standardization of such diverse economic countries may be difficult hence countries with similar economic status region wise  can be compared and studies from countries with similar economic status can help in developing a model for standardization in future.

Are the rationale for, and objectives of, the Systematic Review clearly stated?

Yes

Is the statistical analysis and its interpretation appropriate?

Yes

Are sufficient details of the methods and analysis provided to allow replication by others?

Yes

Are the conclusions drawn adequately supported by the results presented in the review?

Partly

Reviewer Expertise:

Neonatology, birth asphyxia, QI studies in neonatology, neonatal sepsis.

I confirm that I have read this submission and believe that I have an appropriate level of expertise to confirm that it is of an acceptable scientific standard.

# Reviewer report: Reviewer response for version 1

**DOI:** 10.3310/nihropenres.14905.r39460

**Published:** 2026-02-24

**Authors:** Emelda Eberechukwu Chukwu

**Affiliations:** 1 Nigerian Institute of Medical Research, Yaba, Nigeria

This scoping review provides critical economic evaluation of antimicrobial stewardship programs, and reviews the different approaches taken. The authors clearly recognized the extreme heterogeneity across included studies and thoughtfully discussed its implications. The authors failed to conduct a quantitative meta-analysis on the included study, basing their argument that a single summary effect estimate may not meaningfully inform decision-making which I believe is well reasoned.

 The authors made meaningful recommendations including the need to convene an international consensus meeting to standardize outcomes and the proposal to develop an agreed conceptual model linking interventions to process and outcome measures which are really valuable and would provide a practical roadmap for improving comparability and supporting local adaptation of economic models. I especially commend the study for its transparency in acknowledging limitations, particularly the small number of included studies. This openness enhances the credibility of the findings and frames the study as exploratory rather than definitive

 However, a few weaknesses merit attention. Although the heterogeneity of included study was well described, the discussion would have benefited from a more structured categorization of sources of variability (e.g., intervention type, setting, costing perspective, time horizon). Such structure could help readers better understand patterns within the heterogeneity. The implications for software-supported ASPs, although mentioned, are not extensively discussed; more specific guidance for digital or data-driven implementations would strengthen the practical relevance of the paper.

 The authors could have considered outlining a template for reporting economic evaluations of ASPs to encourage consistency. I believe the study made a meaningful contribution by clarifying current gaps and setting a strategic agenda for future research.

Are the rationale for, and objectives of, the Systematic Review clearly stated?

Yes

Is the statistical analysis and its interpretation appropriate?

Not applicable

Are sufficient details of the methods and analysis provided to allow replication by others?

Yes

Are the conclusions drawn adequately supported by the results presented in the review?

Yes

Reviewer Expertise:

Infectious Diseases epidemiology and Genetic basis of Antimicrobial Resistance with focus on AMR surveillance and Antimicrobial stewardship

I confirm that I have read this submission and believe that I have an appropriate level of expertise to confirm that it is of an acceptable scientific standard.

# Reviewer report: Reviewer response for version 1

**DOI:** 10.3310/nihropenres.14905.r34306

**Published:** 2025-01-27

**Authors:** Sajal K Saha

**Affiliations:** 1 Centre for innovation in Infectious Diseases and Immunology Research, Deakin University, Geelong, Victoria, Australia

This is a timely and important scoping review of health economic evaluations of antimicrobial stewardship programmes.

 The objectives are clear but key outcomes could have been defined and described. 

 In the method section, a PRISMA checklist for scoping review can be followed for robust scientific reporting of the study. 

 The review has failed to summarise findings and draw conclusions. However, the authors have provided plausible explanations as the paucity of studies, and heterogeneity of intervention type heterogeneity of method, topic, and context, which is logical and expected.

 The result section could be improved by describing the outcomes of the eligible studies as sub grouped based on the type of studies. 

 The study findings lead to future research directions which have been explained very well.

Are the rationale for, and objectives of, the Systematic Review clearly stated?

Yes

Is the statistical analysis and its interpretation appropriate?

Yes

Are sufficient details of the methods and analysis provided to allow replication by others?

Yes

Are the conclusions drawn adequately supported by the results presented in the review?

Yes

Reviewer Expertise:

Antibiotic stewardship

I confirm that I have read this submission and believe that I have an appropriate level of expertise to confirm that it is of an acceptable scientific standard.
